# Can Differently Stabilized Silver Nanoparticles Modify Calcium Phosphate Precipitation?

**DOI:** 10.3390/ma16051764

**Published:** 2023-02-21

**Authors:** Suzana Inkret, Marija Ćurlin, Kristina Smokrović, Nikolina Kalčec, Nikolina Peranić, Nadica Maltar-Strmečki, Darija Domazet Jurašin, Maja Dutour Sikirić

**Affiliations:** 1 Laboratory for Biocolloids and Surface Chemistry, Division of Physical Chemistry, Ruđer Bošković Institute, Bijenička c. 54, 10000 Zagreb, Croatia; 2 School of Medicine, Catholic University of Croatia, 10000 Zagreb, Croatia; 3 Laboratory for Electron Spin Spectroscopy, Division of Physical Chemistry, Ruđer Bošković Institute, Bijenička c. 54, 10000 Zagreb, Croatia; 4 Institute for Medical Research and Occupational Health, Ksaverska cesta 2, 10000 Zagreb, Croatia

**Keywords:** calcium phosphates, silver nanoparticles, amorphous calcium phosphate, transformation, composites

## Abstract

Calcium phosphates (CaPs) composites with silver nanoparticles (AgNPs) attract attention as a possible alternative to conventional approaches to combating orthopedic implant-associated infections. Although precipitation of calcium phosphates at room temperatures was pointed out as an advantageous method for the preparation of various CaP-based biomaterials, to the best of our knowledge, no such study exists for the preparation of CaPs/AgNP composites. Motivated by this lack of data in this study we investigated the influence of AgNPs stabilized with citrate (cit-AgNPs), poly(vinylpyrrolidone) (PVP-AgNPs), and sodium bis(2-ethylhexyl) sulfosuccinate (AOT-AgNPs) in the concentration range 5–25 mg dm^−3^ on the precipitation of CaPs. The first solid phase to precipitate in the investigated precipitation system was amorphous calcium phosphate (ACP). The effect of AgNPs on ACP stability was significant only in the presence of the highest concentration of AOT-AgNPs. However, in all precipitation systems containing AgNPs, the morphology of ACP was affected, as gel-like precipitates formed in addition to the typical chain-like aggregates of spherical particles. The exact effect depended on the type of AgNPs. After 60 min of reaction time, a mixture of calcium-deficient hydroxyapatite (CaDHA) and a smaller amount of octacalcium phosphate (OCP) formed. PXRD and EPR data point out that the amount of formed OCP decreases with increasing AgNPs concentration. The obtained results showed that AgNPs can modify the precipitation of CaPs and that CaPs properties can be fine-tuned by the choice of stabilizing agent. Furthermore, it was shown that precipitation can be used as a simple and fast method for CaP/AgNPs composites preparation which is of special interest for biomaterials preparation.

## 1. Introduction

Calcium phosphates (CaPs), sparingly soluble salts of phosphoric acid, are of particular interest because of their role in biomineralization and various industrial processes [1]. Importance is further enhanced by the fact that they are found in pathological deposits and on an industrial scale [1,2]. CaPs occur in nature as compounds that differ in Ca/P molar ratio, solubility, and pH range in which they are stable. Of the 12 known non-substituted CaPs, the most abundant are amorphous calcium phosphate (ACP), octacalcium phosphate [OCP, Ca_8_(HPO_4_)_2_(PO_4_)_4_·5H_2_O], calcium hydrogenphosphate dihydrate [DCPD, CaHPO_4_·2H_2_O], calcium deficient apatite [CaDHA, Ca_10−*x*_(HPO_4_)*_x_*(PO_4_)_6−*x*_(OH)_2−*x*_, 0 < *x* < 1], β-tricalcium phosphate [β-TCP, Ca_3_(PO_4_)_2_] and hydroxyapatite [HAP, Ca_10_(PO_4_)_6_(OH)_2_] [3].

The interactions of the nascent CaPs with various types of additives are fundamental to their formation in organisms and are also exploited in the production of materials with well-defined properties [4,5]. In biomineralization, the additives precisely control the nucleation sites, crystal structure, composition, morphology, and orientation of the forming crystals [6,7]. As a result, materials with exceptional properties are formed, that are often unsurpassed by any man-made material [8,9,10].

This motivated the study of the influence of different types of additives on the formation and transformation of CaPs. As a result, the general principles of the CaPs interactions with additives are known, although the studies were conducted under different experimental conditions [4,5,11]. However, this approach has not yet been fully exploited for the rational design of advanced materials [5]. In recent years, various nanomaterials (NMs) have attracted attention as additives in the preparation of CaP-based biomaterials. Two main purposes of using NMs are to improve the mechanical properties of CaPs [12] and/or improve biological properties [13,14], for example, by using nanomaterials with antimicrobial properties. In this sense, silver nanoparticle AgNPs are of particular interest, as silver exhibits a broad spectrum of antimicrobial activity [15]. Silver is unlikely to cause bacterial resistance [16], although there is also evidence to the contrary [17]. This motivated development of silver ion-substituted calcium phosphates [18]. As it was shown that a limited amount of silver can be incorporated in this way [19] and that antimicrobial activity can be increased by using silver nanoparticles (AgNPs) [20], recently attention was turned to the incorporation of AgNPs in CaP-based coatings [21,22], scaffolds [23,24] and dental composites [25,26], as well as to the development of CaP/AgNPs composites. Various methods have been used for the preparation of CaP/AgNPs composites: spray pyrolysis [27,28], pulsed laser irradiation [29], pulsed laser deposition [30], adsorption of AgNPs on pre-prepared CaPs [31,32], mixing AgNPs and biphasic calcium phosphate suspensions [33], sequential treatment of AgNPs with solutions containing calcium or phosphate ions [34], co-precipitation of AgNPs and CaPs [35,36,37], precipitation at elevated temperatures [38].

The preparation of biomaterials by precipitation at low temperatures is considered an economically and environmentally friendly synthetic route [39,40]. Moreover, the activity of biologically active molecules can be easily preserved in this way, and the coating of complex shapes is possible. However, to the best of our knowledge, the precipitation of CaPs at low temperatures in the presence of AgNPs has not yet been studied. From the fundamental point of view, such a procedure could provide an additional possibility to control the process by using differently stabilized AgNPs.

To fill this void, in this study we investigated the precipitation of calcium phosphate in the presence of AgNPs stabilized with citrate (cit-AgNPs), poly(vinylpyrrolidone) (PVP-AgNPs), and sodium bis(2-ethylhexyl) sulfosuccinate (AOT-AgNPs). A precipitation system was chosen in which the formation of the precipitate proceeds through an amorphous precursor to be able to determine the effects on the formation and properties of amorphous (ACP) and crystalline CaPs. The results obtained indicate that subtle differences in the properties of the formed CaPs were caused by differently stabilized AgNPs. They also confirm that precipitation at room temperature can be used as a simple and rapid method for the preparation of CaP/AgNPs composites.

## 2. Materials and Methods

### 2.1. Materials

The following analytical grade chemicals were used: silver nitrate (AgNO_3_), sodium citrate dihydrate (C_6_H_5_Na_3_O_7_·2H_2_O, cit), poly(vinylpyrrolidone) ((C_6_H_9_NO)_x_, PVP) with average molecular weight *M*_r_ = 40,000 g mol^−1^, sodium bis(2-ethylhexyl) sulfosuccinate (C_20_H_37_NaO_7_S, AOT), glucose (C_6_H_12_O_6_), ammonium (NH_3_), calcium chloride dihydrate (CaCl_2_ × 2H_2_O), sodium hydrogenphosphate (Na_2_HPO_4_), sodium hydroxide (NaOH), hydrochloric acid (HCl). All chemicals were purchased from Sigma Aldrich, Darmstadt, Germany. Ultrapure water (UPW, conductivity 0.5 μS cm^−1^, Hydrolab HLP 10 UV, Straszyn, Poland) was used for all experiments.

### 2.2. Synthesis of Silver Nanoparticles

#### 2.2.1. Citrate-Coated Silver Nanoparticles

Cit-AgNPs were synthesized according to the modified method described previously [41,42]. Briefly, 2.2 mL of a 90 × 10^−3^ mol dm^−3^ solution of silver nitrate was added to 195 mL of ultrapure water. The solution was brought to boiling under rapid stirring and reflux. Immediately after boiling, 2.27 mL of a 1% (*w/v*) sodium citrate dihydrate solution was rapidly added to the reaction mixture. The reaction mixture was stirred continuously at 90 °C for about 15 min. After the color changed from colorless to yellow, the reaction mixture was kept under rapid stirring for 3 h. The freshly prepared cit-AgNPs suspension was washed twice with ultrapure water by centrifugation at 15,790× *g* for 20 min. The purified cit-AgNPs were resuspended in ultrapure water and stored in the dark at 4 °C until use.

#### 2.2.2. AOT and PVP Coated AgNPs

AOT-AgNPs and PVP-AgNPs were prepared according to the modified method of Vinković Vrček et al. [43,44], using glucose instead of NaBH_4_ as a reducing agent. The syntheses of AOT-AgNPs and PVP-AgNPs were performed at room temperature and 40 °C, respectively. To the aqueous solution of stabilizing agent (193 mL of 5 × 10^−3^ mol dm^−3^ AOT or 192.7 mL of 0.3 % PVP) at the appropriate temperature, 2.22 mL of a 90 × 10^−3^ mol dm^−3^ AgNO_3_ solution was added with stirring. Next, 0.133 mL of a 35 % NH_3_ solution was added, followed by the addition of 4 mL of 0.5 mol dm^−3^ glucose solution at a rate of approximately 1 drop/s. Finally, 0.6 mL of 1 mol dm^−3^ NaOH was added. The obtained suspensions were kept at synthesis temperature for another 30 min under stirring. The purified AgNPs were obtained and stored for further use as described for cit-AgNPs.

### 2.3. Precipitation System

The cationic (CaCl_2_) and anionic reactant (Na_2_HPO_4_) stock solutions were prepared by dissolving the required amount of analytical-grade chemicals in ultra-pure water. Before the preparation of stock solutions, chemicals were dried overnight in a desiccator over silica gel. Subsequently, the pH of the Na_2_HPO_4_ stock solution was adjusted to 7.4 with HCl.

The anionic and cationic reactant solutions were prepared by diluting the corresponding stock solutions to the concentration *c* = 8 · 10^−3^ mol dm^−3^. The required amount of AgNPs suspension was added to the anionic reactant solution during the dilution of the stock solution. If necessary, the pH of the anionic reactant solution was readjusted.

The precipitation systems were prepared by rapidly mixing 200 cm^3^ of anionic and cationic reactant solutions, resulting in the initial reactant concentrations *c*(CaCl_2_) = *c*(Na_2_HPO_4_) = 4 · 10^−3^ mol dm^−3^ and *c*(AgNPs) = 5, 10, and 25 mg dm^−3^ at pH = 7.4. Precipitation experiments were performed in a double-walled vessel at 25 ± 0.1 °C without additional stirring. The reaction vessel was kept in the dark.

The progress of precipitation was followed by monitoring the pH (913 pH meter, Metrohm, Herisau, Switzerland) of the precipitation system. Based on the pH vs. time curves, the formed precipitates were filtered at aging times corresponding to the formation of amorphous and crystalline phases. The precipitates were filtered through a 0.45 μm membrane filter, washed thoroughly with ultrapure water, and once with ethanol. Subsequently, they were dried in a nitrogen stream and stored in the dark in a desiccator until further analysis.

Saturation indices, defined as the logarithm of the ratio of ion activity product and solubility constant of each solid phase, were calculated from the initial total concentrations of the reactants using VMINTEQ 3.1 (available at http://vminteq.lwr.kth.se/download/ (accessed on 21 April 2021).

### 2.4. Characterization Methods

#### 2.4.1. Powder X-ray Diffraction

Powder X-ray Diffraction (PXRD) patterns of the precipitates were obtained on a Panalytical Aeris Research Edition (Malvern Pananalytical, Malvern, Worcestershire, UK) in Bragg–Brentano geometry using CuKα radiation. Patterns were recorded in an angular scan range of 5° to 70° 2*θ* with a step size of 0.02° 2*θ* and a scan rate of 1° min^−1^.

#### 2.4.2. Fourier Transform Infrared Spectroscopy

Fourier Transform Infrared (FTIR) spectra of the precipitates were obtained by an FTIR spectrometer equipped with an attenuated total reflection module (Tensor I, Bruker, Ettlingen, Germany). The spectra were recorded in the range from 4000−450 cm^−1^ with a resolution of 1 cm^−1^ and are average of 16 scans.

The first- and second-order differentiated FTIR spectra in the 1200–450 cm^−1^ range were obtained following the procedure described by Uskoković [45] using a manual differentiation routine in Origin Pro 2021b.

#### 2.4.3. Atomic Absorption Spectroscopy

To determine the silver concentration in the AgNPs suspensions, the samples were dissolved in 10% (*v/v*) HNO_3_. Silver concentrations were determined using a graphite furnace atomic absorption spectrometer (GFAAS) (Perkin Elmer AAnalyst 600, Perkin Elmer, Shelton, CT, USA) with Zeeman background correction. A silver standard solution (1000 mg dm^−3^ in 5% HNO_3_), purchased from Merck (Darmstadt, Germany), was used for calibration.

#### 2.4.4. UV-Vis Spectroscopy

The UV-Vis spectra of the aqueous AgNPs suspensions were obtained by Carry 60 UV-Vis spectrophotometer (Agilent, Santa Clara, CA, USA). The presence of a surface plasmon resonance peak was used to confirm the formation of AgNPs.

#### 2.4.5. Dynamic and Electrophoretic Light Scattering

The size distribution and zeta potential of AgNPs in an anionic reactant solution were determined by dynamic (DLS) and electrophoretic light scattering (ELS) using a photon correlation spectrophotometer with a 532 nm “green” laser (Zetasizer Nano ZS, Malvern Instruments, Worcestershire, UK). For the DLS measurements, the intensity of the scattered light was detected at an angle of 173°. To avoid overestimation due to the scattering of larger particles, the hydrodynamic diameter (*d*_h_) was determined as the value of the peak maximum of the size volume distribution function. The zeta potential (*ζ*) was calculated from the measured electrophoretic mobility using Henry’s equation and the Smoluchowski approximation. To determine the size distribution, each sample was measured six times, while for determining zeta potential the samples were measured three times. Representative data are shown. Data processing was performed using Zetasizer Software 8.02 (Malvern Instrument, Worcestershire, UK). All measurements were conducted at 25.0 ± 0.1 °C.

#### 2.4.6. Thermogravimetric Analysis

Thermogravimetric analysis (TGA) of the precipitates obtained after 1 h of reaction time was carried out on an STA 449 F5 Jupiter thermal analyzer (Netzsch, Bayern, Germany) and Mettler TG 50 thermobalance (Mettler Toledo Corp., Zürich, Switzerland) equipped with a TC 10 TA processor. The measurements were performed in the stream of air and at a heating rate of 10 K min^−1^.

#### 2.4.7. Electron Paramagnetic Spectroscopy (EPR)

Electron paramagnetic resonance (EPR) measurements were performed using the Bruker Magnettech ESR5000 benchtop EPR spectrometer operating at X-band frequencies with a resonant microwave frequency of 9.4 GHz. The magnetic field was modulated at 100 kHz with a peak-to-peak amplitude of 0.1 mT. The temperature was controlled using a variable temperature controller for liquid nitrogen (TCH04). Manganese, Mn^2+^ in ZnS, (Bruker module E8000137) was used as a field standard to control and calibrate the magnetic field axis. All measurements were performed at room temperature. No EPR signal was detected in the empty EPR tube or the non-irradiated samples. To create paramagnetic centers, all samples were irradiated in the presence of air at room temperature with gamma rays from the panoramic Co-60 irradiator of the Laboratory of Radiation Chemistry and Dosimetry at the Ruđer Bošković Institute [46]. The total dose was 25 kGy, which is considered the ‘gold standard’ for the sterilization of food, medical devices, and other healthcare products [47,48]. Radiation-induced free radicals have been used as molecular probes for further EPR analysis of samples.

#### 2.4.8. Transmission Electron Microscopy (TEM)

Transmission electron microscopy images of AgNPs and amorphous precipitates were acquired using a JEOL JEM 1010 transmission electron microscope (JEOL, Tokio, Japan) operated at 80 kV. A drop of the suspension was placed on the copper grid covered with the hollow Formvar membrane. The excess solution was removed with filter paper and the remaining precipitate was washed three times with a drop of UPW. The samples were dried in the stream of nitrogen and stored in the dark in a desiccator until analysis.

Primary size distributions of AgNPs and spherical ACP particles were determined using Image J 1.48 v image analysis software (freely available at http://imagej.nih.gov/ij/ (accessed on21 April 2021). The size of at least 30 particles was measured for each sample.

#### 2.4.9. Scanning Electron Microscopy (SEM)

For imagining crystalline precipitate field emission scanning electron microscope (FE-SEM; JEOL JSM-7000 F microscope, JEOL, Tokyo, Japan) was used. For SEM analysis a small amount of dried precipitate was placed on a sample holder covered with carbon adhesive. The excess precipitate was removed with a light stream of nitrogen.

## 3. Results and Discussion

### 3.1. Characterization of AgNPs

**Figure 1:**
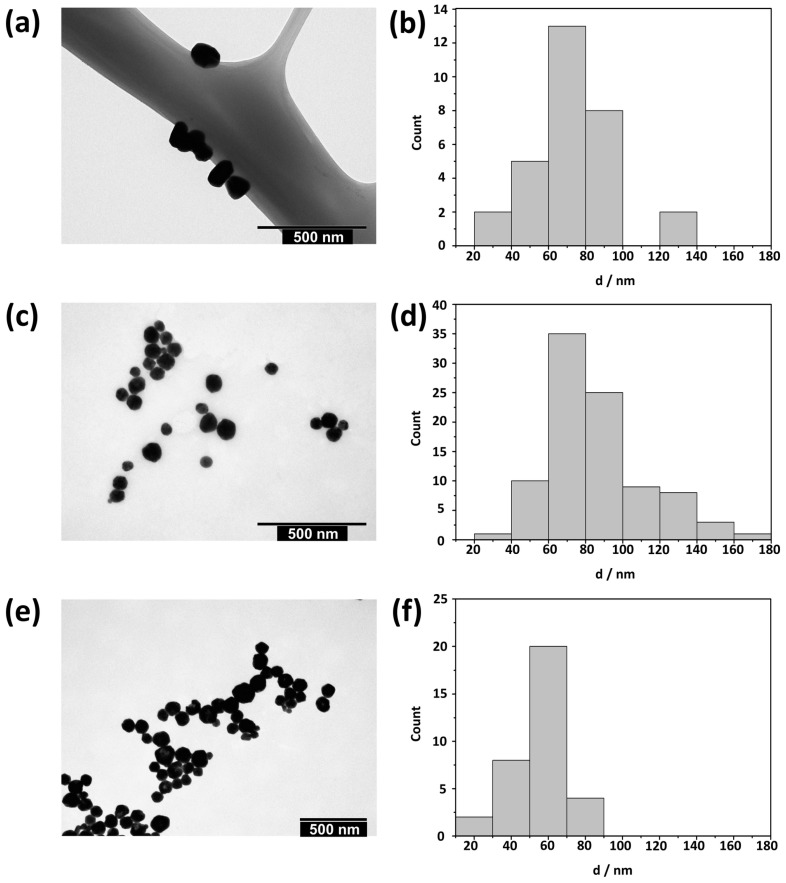
(**a**,**c**,**e**) TEM micrographs and (**b**,**d**,**f**) corresponding size distributions of silver nanoparticles stabilized with (**a**,**b**) citrate, (**c**,**d**) poly(vinylpyrrolidone), and (**e**,**f**) sodium bis(2-ethylhexyl) sulfosuccinate.

**Figure 2:**
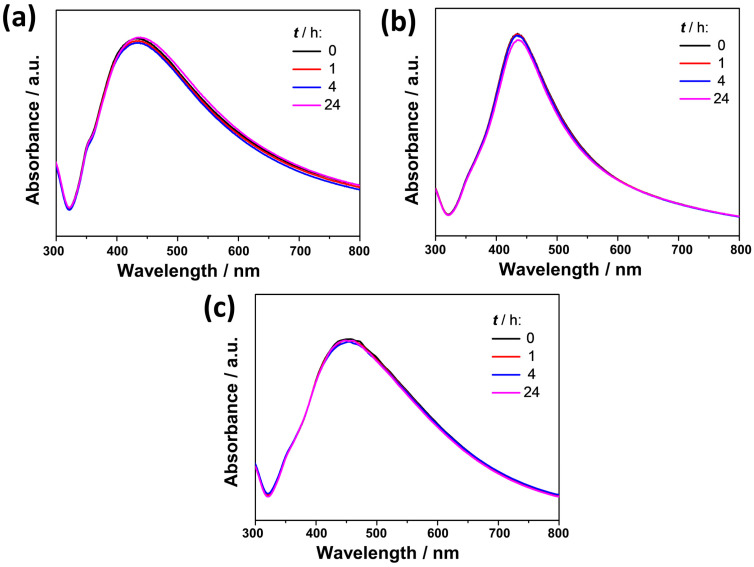
UV-Vis spectra of silver nanoparticles (AgNPs), stabilized with (**a**) citrate (cit-AgNPs), (**b**) poly(vinylpyrrolidone) (PVP-AgNPs), and (**c**) sodium bis(2-ethylhexyl) sulfosuccinate (AOT-AgNPs). (*γ* (AgNPs) = 50 mg dm^−3^) in UPW at 25 °C recorded at different time periods.

**Figure 3:**
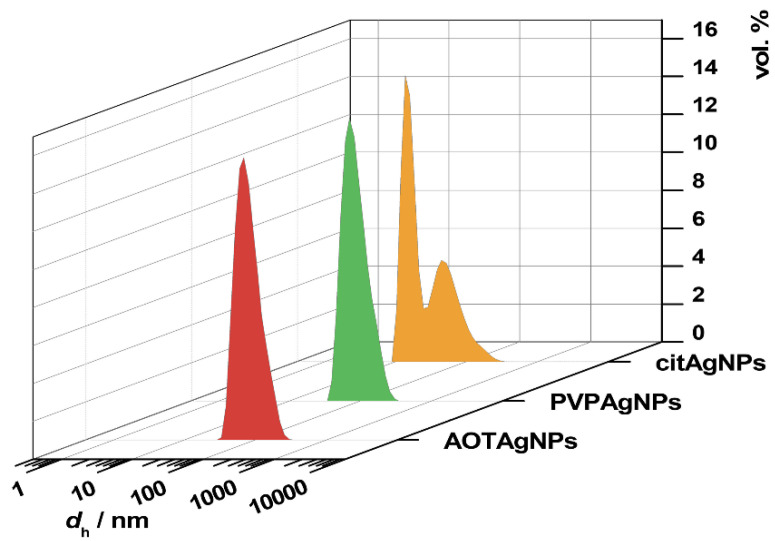
Representative volume size distribution of silver nanoparticles (AgNPs), stabilized with citrate (cit-AgNPs), poly(vinylpyrrolidone) (PVP-AgNPs), sodium bis(2-ethylhexyl) sulfosuccinate (AOT-AgNPs) suspended in anionic reactant solution (*c*(Na_2_HPO_4_) = 8 · 10^−3^ mol dm^−3^, *γ* (AgNPs) = 50 mg dm^−3^, pH 7.4) at 25 °C. *d*_h_—hydrodynamic diameter.

The freshly prepared AgNPs were imaged by TEM (Figure 1) and characterized by UV-Vis spectroscopy (Figure 2), DLS, and ELS measurements (Table 1 and Figure 3).

TEM micrographs revealed the presence of quasi-spherical and triangular cit-AgNPs (Figure 1a). In addition, sporadically, longer rod-like particles were observed, consistent with previous studies [42]. The average size of cit-AgNPs was 75.1 ± 23.2 nm (Figure 1b). PVP-AgNPs formed as polyhedral particles with an average size of 85.2 ± 26.0 nm (Figure 1c,d). A similar morphology was observed for AOT-AgNPs (Figure 1e), but they had a smaller average size, 55.42 ± 15.0 nm, and a narrower distribution than PVP-AgNPs (Figure 1f).

The UV-Vis spectra of the synthesized AgNPs are shown in Figure 2. The presence of the SPR peak at 433, 435, and 450 nm in the spectra of cit-AgNPs, PVP-AgNPs, and AOT-AgNPs, respectively, confirmed the formation of nanoparticles [49]. In addition to the position, the width of the plasmon band also varied depending on the type of particle. However, in all cases the intensity and position of the SPR peak didn’t change during 24 h, indicating good stability of all the AgNPs prepared.

To determine a possible aggregation of the AgNPs in the anionic reactant solution, the size distribution and the zeta potential of the nanoparticles were determined (Figure 3 and Table 1). DLS measurements revealed a bimodal size distribution of the cit-AgNPs. A dominant population of particles with an average *d*_h_ value of 15.9 nm and a population of larger particles with an average *d*_h_ value of 64.4 nm was detected. Monomodal particle size distributions with similar average *d*_h_ values of 89.9 nm and 84.8 nm, were observed in the suspensions of PVP-AgNPs and AOT-AgNPs in an anionic reactant solution, respectively.

The low aggregation of AgNPs in an anionic reactant solution might be due to their relatively large negative zeta-potential ranging from −24.5 mV for PVP-AgNPs to −59.9 mV for AOT-Ag NPs (Table 1).

### 3.2. Influence of Silver Nanoparticles on Precipitation of Calcium Phosphates

To investigate the influence of AgNPs on the precipitation of CaPs, a precipitation system was chosen in which crystalline CaPs are formed via the formation of the amorphous precursor ACP.

#### 3.2.1. Influence of AgNPs on Amorphous Calcium Phosphate

The precipitation of CaPs is accompanied by a change in the pH of the precipitation system [50,51,52]. In the pH range 4.5–9.5, where ${\mathrm{HPO}}_{4}^{2-}$ and $H_{2}{\mathrm{PO}}_{4}^{-}$ are the dominant phosphate species, the formation of CaPs can be represented by the equation [53]:

(1) $$\begin{array}{cc} x{\mathrm{Ca}}^{2+}+y{\mathrm{HPO}}_{4}^{2-} & +zH_{2}{\mathrm{PO}}_{4}^{-}+wH_{2}O\\ & ⇄{\;\mathrm{Ca}}_{x}{\left({\mathrm{HPO}}_{4}\right)}_{u}{\left({\mathrm{PO}}_{4}\right)}_{v}{\left(\mathrm{OH}\right)}_{t}+\left(y+2z+w-u\right)H^{+} \end{array}$$

**Figure 4:**
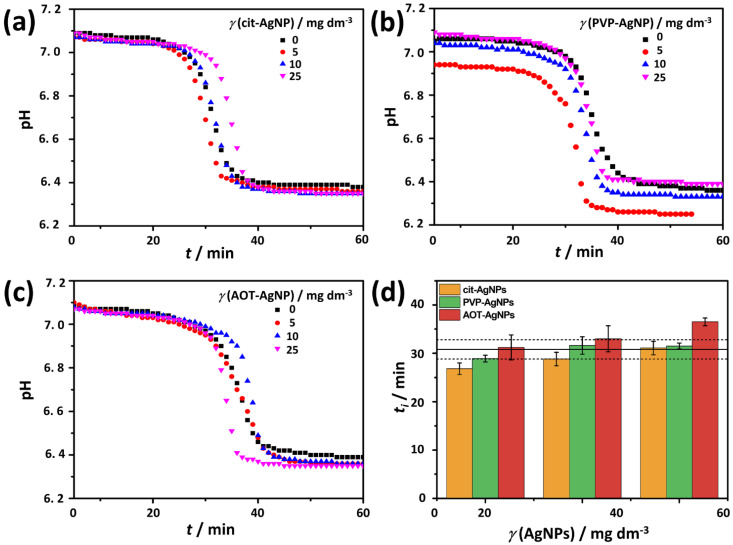
Representative pH vs. time curves (**a**–**c**) and corresponding average induction time for crystalline phase precipitation (*t*_i_) with corresponding standard deviation (**d**) obtained in the control system (*γ*(AgNPs) = 0 mg dm^−3^) and in the presence of different concentrations of silver nanoparticles (AgNPs) stabilized with (**a**) citrate (cit-AgNPs), (**b**) poly(vinylpyrrolidone) (PVP-AgNPs), and (**c**) sodium bis(2-ethylhexyl) sulfosuccinate (AOT-AgNPs). In figure (**d**) full and dashed lines represent the average induction time and corresponding standard deviation in the control system, respectively. *c*(CaCl_2_) = *c*(Na_2_HPO_4_) = 4 · 10^−3^ mol dm^−3^, pH 7.4, 25 °C.

The change in pH reflects the different stages of the precipitation process and allows the progress of the precipitation process to be followed, at least semi-quantitatively [50,51,52]. The representative pH vs. time curves obtained in the control system and in systems with different concentrations of the investigated AgNPs are shown in Figure 4a–c. In all precipitation systems studied, sigmoidal curves were obtained, reflecting three different stages of the precipitation process. The first stage, associated with a slight decrease in pH, corresponds to the formation of ACP. According to Du et al. [54], such a pH change indicates that the formation of ACP occurs through a ligand substitution reaction in which water molecules in the Ca^2+^ coordination sphere are replaced by partially protonated phosphate ions. In the second stage, an abrupt drop in pH is associated with secondary precipitation of the crystalline phase on the already formed ACP. The final stage of the precipitation process, solution-mediated growth, and phase transformation are also associated with a slight change in pH [50,52,55,56,57]. The similar shape of the curves obtained may indicate that the AgNPs did not cause a change in the pathway of calcium phosphate formation. This is consistent with previous studies that have shown that the precipitation pathway of CaP is not influenced by different classes of additives such as small ions [56,58,59], liposomes [60], phosphorylated osteopontin peptides [55], cationic and anionic polyelectrolytes [50], and TiO_2_ nanomaterials of different dimensionality [61,62]. On the other hand, data on the influence of amino acids are contradictory [58,61], while surfactants have been shown to affect the formation pathway [52].

The stability of the ACP under certain conditions can be assessed from the pH vs. time curves, by determining the induction time for the formation of the crystalline phase (*t*_i_) i.e., the time elapsed between the start of the reaction and the start of the secondary precipitation of the crystalline phase, as indicated by an abrupt change in pH in the second stage (Figure 4a–c) [50,52]. The *t*_i_ is determined from the intersection of the tangents drawn at the first two parts of the pH vs. time curve [50]. The longer it is, the more stable ACP is considered to be [50,59]. In the control system, the average *t*_i_ was 30.8 ± 2 min (Figure 4d). In all cases, the average *t*_i_ increased with increasing AgNPs concentration. Compared to the control system, a significantly shorter *t*_i_ was obtained in the presence of the lowest concentration of cit-AgNPs studied, indicating the promotion of ACP transformation. In contrast, a longer *t*_i_ was obtained only in the presence of the highest applied concentration of AOT-AgNPs, indicating stabilization of ACP. Interestingly, *t*_i_ increased at all AgNPs concentrations studied in the order cit-AgNPs < PVP-AgNPs < AOT-AgNPs, indicating a subtle influence of surface coating. A previous study showed that TiO_2_ nanoparticles (TiNPs) at low concentrations, comparable to the concentrations of AgNPs investigated in this study, also prolonged ACP transformation. However, at higher concentrations, the transformation was accelerated. This was attributed to the dominant formation of ACP on TiNPs at higher concentrations [61]. Interestingly, a previous study on the effect of citrate on ACP transformation showed that citrate has the opposite effect, i.e., it stabilizes ACP [59]. However, it should be noted that in that case the citrate was dissolved, and not bound to the surface as in the case of cit-AgNPs.

**Figure 5:**
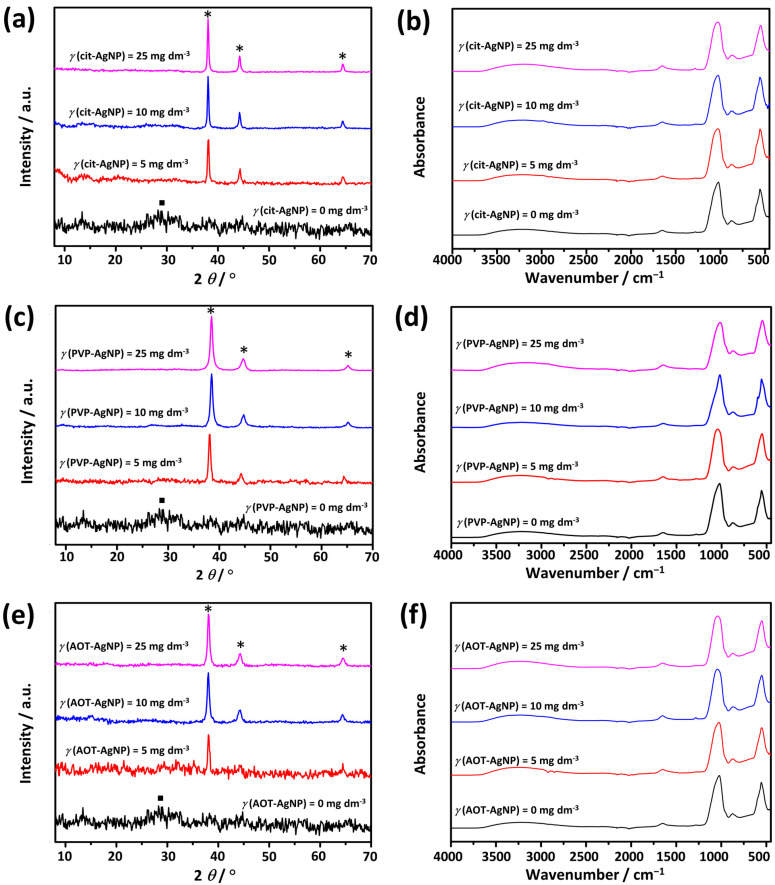
PXRD patterns (**a**,**c**,**e**) and FTIR spectra (**b**,**d**,**f**) of precipitates obtained after 10 min reaction time in the control system and in the presence of silver nanoparticles (AgNPs) stabilized with (**a**,**b**) citrate (cit-AgNPs), (**c**,**d**) poly(vinylpyrrolidone) (PVP-AgNPs), and (**e**,**f**) sodium bis(2-ethylhexyl) sulfosuccinate (AOT-AgNPs). *c*(CaCl_2_) = *c*(Na_2_HPO_4_) = 4 · 10^−3^ mol dm^−3^, pH 7.4, 25 °C. ■—amorphous calcium phosphate, *—silver nanoparticles.

**Figure 6:**
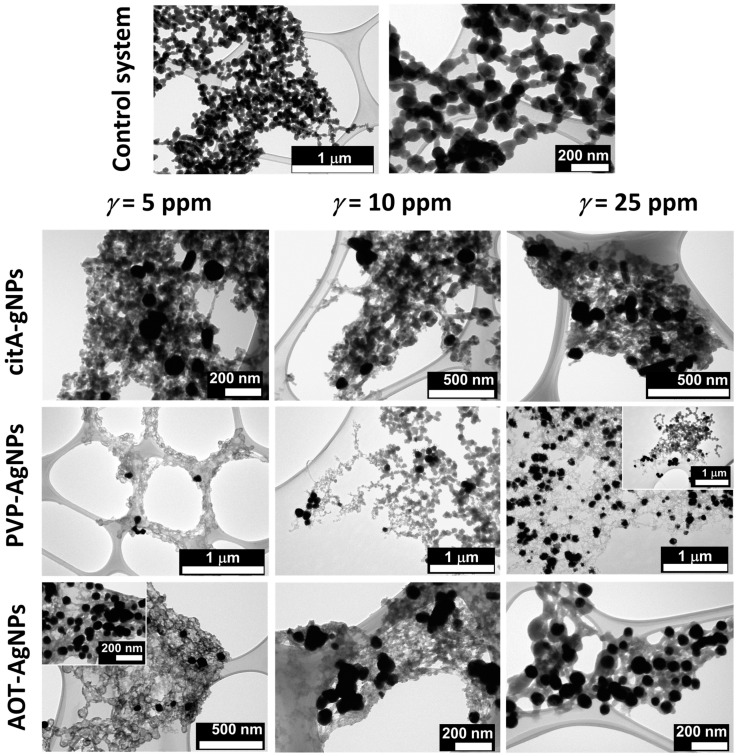
TEM micrographs of precipitates obtained after 10 min reaction time in the control system and in the presence of different concentrations of silver nanoparticles (AgNPs) stabilized with citrate (cit-AgNPs), poly(vinylpyrrolidone) (PVP-AgNPs), and sodium bis(2-ethylhexyl) sulfosuccinate (AOT-AgNPs). *c*(CaCl_2_) = *c*(Na_2_HPO_4_) = 4 · 10^−3^ mol dm^−3^, pH 7.4, 25 °C.

To determine the influence of AgNPs on the properties of ACP, the precipitates formed after 10 min of reaction time were analyzed by PXRD, FTIR (Figure 5), and TEM (Figure 6). In the PXRD pattern of the precipitate, formed in the control system after 10 min, a broad amorphous peak of low intensity was observed at 2*θ* 25.0°–33.5° (Figure 5). Such a peak is characteristic of ACP [27,63,64]. In the PXRD patterns obtained in the presence of different concentrations of AgNPs, the amorphous peak characteristic of ACP was observed only in the presence of the lowest concentration of cit-AgNPs, but not in the PXRD patterns of the precipitates formed in other systems with AgNPs, due to the high intensity of the peaks characteristic of silver observed at 2*θ* 38.1°, 44.2° and 64.50° corresponding to the (111), (200) and (220) reflections of silver (ICCD File No 04-0783), respectively (Figure 5a,c,e).

The FTIR spectra (Figure 5) confirmed that ACP was formed in all precipitation systems. In the FTIR spectrum of ACP formed in the control system, vibrational bands characteristic of phosphate groups were observed at 1024 cm^−1^ (*ν*_3_ asymmetric stretching mode of ${\mathrm{PO}}_{4}^{3-}$ group), 875 cm^−1^ (${\mathrm{HPO}}_{4}^{2-}$ group), and 553 cm^−1^ (*ν*_4_ bending mode of ${\mathrm{PO}}_{4}^{3-}$ group). Water bands were observed at 3677–2789 cm^−1^ (broad band), and 1651 cm^−1^ [63,65]. The absence of a multiplet structure of the phosphate bands at 1024 cm^−1^ and 553 cm^−1^ is characteristic of an amorphous phase [45,63,65]. A more detailed analysis of the phosphate band region was performed using first- and second-order derivative spectra (Figure S1). While the position of the bands can be determined more precisely using the first-order derivative, the second-order derivative can be used to distinguish overlapping features [66]. The largest shift in the maximum of the band characteristic of the asymmetric stretching mode of the ${\mathrm{PO}}_{4}^{3-}$ group to a higher wavenumber by 25 cm^−1^ was observed at the two highest concentrations of AOT-AgNPs and the lowest concentration of PVP-AgNPs, indicating somewhat different influences of these two types of nanoparticles (Figure S1). Spectral features observed in the second-order derivative spectra in 1150–1030 cm^−1^ and 650–575 cm^−1^ regions indicated the onset of ACP transformation (Figure S1). Similar features were previously observed during ACP transformation to HAP [45].

**Figure 7:**
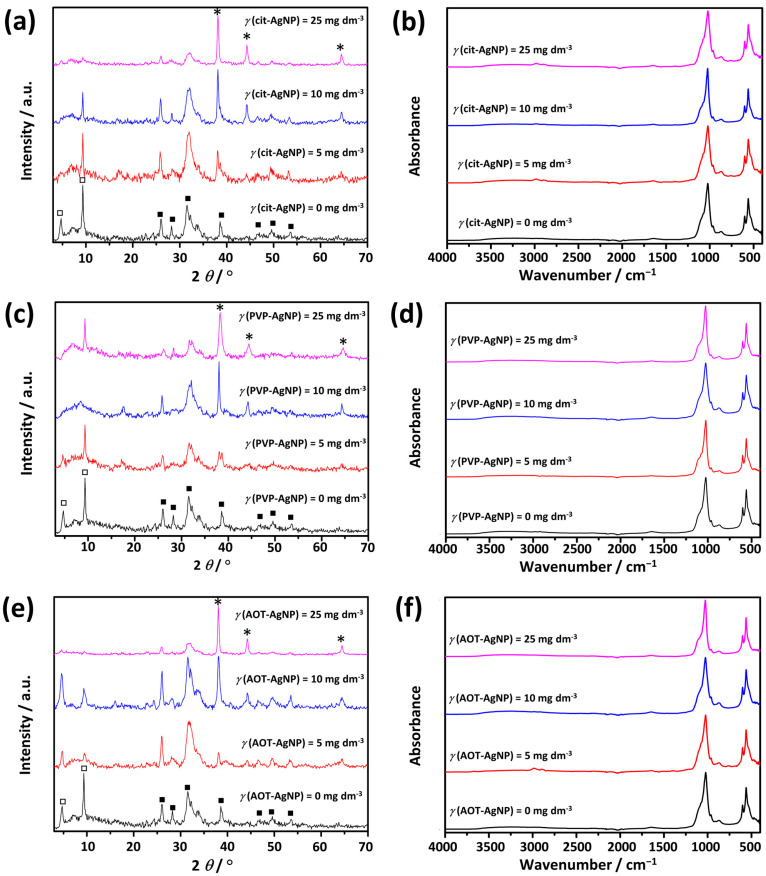
PXRD patterns (**a**,**c**,**e**) and FTIR (**b**,**d**,**f**) spectra of precipitates obtained after 60 min reaction time in the presence of silver nanoparticles (AgNPs) stabilized with (**a**,**b**) citrate (cit-AgNPs), (**c**,**d**) poly(vinylpyrrolidone) (PVP-AgNPs), and (**e**,**f**) sodium bis(2-ethylhexyl) sulfosuccinate (AOT-AgNPs). *c*(CaCl_2_) = *c*(Na_2_HPO_4_) = 4 · 10^−3^ mol dm^−3^, pH 7.4, 25 °C. □—octacalcium phosphate, ■—calcium deficient hydroxyapatite, *—silver nanoparticles.

TEM micrographs (Figure 6) showed that chain-like aggregates of spherical particles typical of ACP [50,64] formed in the control system. The average size of the individual ACP particles was 75.6 ± 19.2 nm. In the presence of AgNPs, two types of ACP morphologies were observed, depending on the type of AgNPs and their concentration. In the case of cit-AgNPs, a dense gel-like phase was formed at all concentrations. Such a morphology was also observed previously for ACP formed in different conditions [52,58,67,68]. It is worth noting, that in the presence of dissolved citrate, chain-like aggregates of ACP particles with rougher surfaces compared to the control were obtained [59]. The different effects on morphology could be a consequence of different citrate concentrations, as well as of the different states of the citrate, i.e., dissolved or bound to the AgNPs. With increasing cit-AgNPs concentration, the granular structure of the dense phase decreased. In the presence of PVP-AgNPs, chain-like aggregates of irregular spherical particles were observed at all concentrations, while the gel-like phase was observed only at the highest concentration studied. In contrast, in the presence of AOT-AgNPs, the chain-like aggregates of spherical particles were observed only at the lowest concentration studied, whereas the gel-like phase was observed at all investigated concentrations (Figure 7). The spherical ACP particles formed in the presence of PVP-AgNPs (67.6 ± 16.2 nm) and AOT-AgNPs (76.7 ± 21.4 nm and 66.1 ± 17.2 nm, at 10 and 25 mg dm^−3^, respectively) were similar in size to those in the control system. In both cases, the onset of their transformation to the gel phase was visible. The obtained result indicated a possible method of controlling ACP morphology by the selection of an AgNPs stabilizing agent.

Interestingly, in all cases, the NPs were distributed throughout the ACP, and single particles embedded in ACP were mostly observed. This indicates the potential of obtaining the material in which the NPs are dispersed throughout the material, the lack of which was cited as one of the major drawbacks for existing CaP/AgNPs composites [69]. To the best of our knowledge, Keskar et al. [27] were the only ones to study ACP-AgNPs composites. They obtained spherical ACP particles with smaller AgNPs incorporated into the particles and/or their surface by spray pyrolysis.

#### 3.2.2. Influence of AgNPs on the Properties of Crystalline Phase

According to the supersaturation calculation, the control precipitation system was supersaturated to different CaPs phases (ACP, β-TCP, OCP, DCPD, HAP). PXRD and FTIR analyses were performed to determine the composition of the precipitates formed (Figure 7). The PXRD pattern of the control system (Figure 7a) contained two low angle peaks at 2*θ* 4.7° and 9.3° corresponding to (100) and (200) reflections of OCP (JCPDS card 26-1056). In addition, prominent apatitic peaks at 2*θ* 26.0° and 31.8°, as well as lower intensity peaks at 2*θ* 28.1°, 39.3°, 46.4°, 49.6° and 53.3° were observed. The shape and width of apatitic peaks indicated the possibility of the formation of poorly crystalline CaDHA [63,70]. It should be noted, that compared to our previous studies on the effect of TiNMs CaP precipitation, [61,62] in which the same reactant concentrations were used, the reaction volume was increased tenfold, resulting in the difference in precipitate composition.

Characteristic phosphate and water vibration bands were observed in the FTIR spectra of the control system (Figure 7b). Phosphate bands were observed at 1303 cm^−1^ (${\mathrm{HPO}}_{4}^{2-}$ bending), 1091 cm^−1^ (*ν*_3a_ triply degenerated asymmetric stretching mode of ${\mathrm{PO}}_{4}^{3-})$, 1020 cm^−1^ (*ν*_3c_ triply degenerated asymmetric stretching mode of ${\mathrm{PO}}_{4}^{3-}$), 964 cm^−1^ (*ν*_1_ nondegenerated symmetric stretching mode of ${\mathrm{PO}}_{4}^{3-}$), 912 cm^−1^ and 869 cm^−1^(${\mathrm{HPO}}_{4}^{2-}$ stretching), 597 cm^−1^ (*ν*_4a_ triply degenerated bending mode of ${\mathrm{PO}}_{4}^{3-}$), and 560 cm^−1^ (*ν*_4b_ triply degenerated bending mode of ${\mathrm{PO}}_{4}^{3-}$. The water bands were observed at 3627–2884 cm^−1^ (broad band) and 1637 cm^−1^ [71,72,73]. Although there is a great similarity between the FTIR spectra of apatites and OCP exists, it is considered that the bands at around 1195 cm^−1^ and 916 cm^−1^ correspond to the vibrations of ${\mathrm{HPO}}_{4}^{2-}$ groups in the OCP lattice, can be used to distinguish between CaDHA and OCP in the FTIR spectra [73]. In the spectrum of the control system, the band at 912 cm^−1^ was detected, but not the band at around 1195 cm^−1^, indicating that the amount of OCP was smaller than that of CaDHA. Also, the hyperfine structure of the phosphate bands in the 1200–1000 cm^−1^ region, which is characteristic of OCP, refs. [73,74] was not observed, confirming that OCP is present in a small amount.

The PXRD patterns of the precipitates prepared in the presence of AgNPs (Figure 7a,c,e) in addition to CaPs peaks, contained Ag peaks at around 2*θ* 38.0°, 44.3°, and 64.4°. It was found that the relative intensity of OCP peaks at around 2*θ* 4.7° and 9.5° varied depending on the type of AgNPs and their concentration. Both these peaks were not observed at the highest applied AOT-AgNPs concentration and 10 mg dm^−3^ concentration of PVP-AgNPs. This indicates possible variations in the amount of precipitated OCP, i.e., possible inhibition of OCP formation. Studies on the formation of calcium phosphates in the presence of various additives have shown that additives can affect the composition of the precipitates formed in two ways. In precipitation systems where several phases can form, the additive can effectively adsorb to the nuclei of a particular phase, inhibiting its formation. Another possibility is that the additive acts as an efficient heteronucleus for one of the forming phases, promoting its formation and indirectly inhibiting the formation of other phases [4,75].

Analysis of the first-order derivative FTIR spectra in the 1200–450 cm^−1^ phosphate band region (Figure S2) revealed no significant changes in the position of the bands. However, the second-order derivative spectra showed the shape change in the region 1005–975 cm^−1^ at the highest investigated concentrations of PVP-AgNPs and AOT-AgNPs, as well as at 10 mg dm^−3^ of cit-AgNPs.

**Figure 8:**
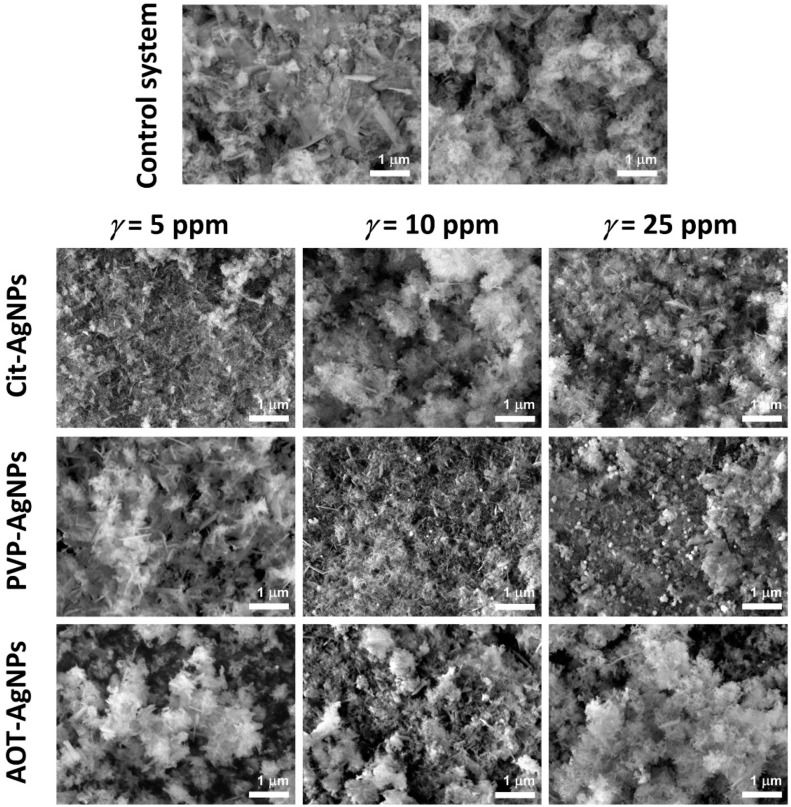
SEM micrographs of precipitates obtained after 60 min reaction time in the control system and in the presence of different concentrations of silver nanoparticles (AgNPs) stabilized with citrate (cit-AgNPs), poly(vinylpyrrolidone) (PVP-AgNPs), and sodium bis(2-ethylhexyl) sulfosuccinate (AOT-AgNPs). *c*(CaCl_2_) = *c*(Na_2_HPO_4_) = 4 · 10^−3^ mol dm^−3^, pH 7.4, 25 °C.

SEM analysis of the precipitates formed in the control system and in the presence of different concentrations of AgNPs confirmed that a mixture of different CaPs phases was formed (Figure 8). Two different morphologies were observed in all systems. Spherical aggregates of thin, irregular, leaf-like crystals are characteristic of CaDHA, [76] while single plate-like crystals are characteristic of OCP [50,77]. In addition, individual AgNPs can be observed both embedded in the CaDHA crystal aggregates and adsorbed on their surface. It is difficult to comment on the changes in OCP crystal morphology, because most crystals in the precipitates formed in the presence of AgNPs are oriented with their largest face perpendicular to the substrate. However, compared to the control system, they appear smaller in size and less developed. With increasing AgNPs concentrations, the morphological changes of OCP became more evident. The changes in the morphology of CaDHA depended on the type of AgNPs. In the presence of lower concentrations of cit-AgNPs, the spherical aggregates were denser and more fused together. With increasing concentration, more distinct spherical aggregates were observed, which were smaller at the highest concentration studied than in the control system. The morphology of the precipitate obtained at the lowest concentration of PVP-AgNPs was similar to that of the control system. With increasing concentrations of PVP-AgNPs, smaller crystals, and fused spherical aggregates were observed. In the presence of AOT-AgNPs, the spherical aggregates were the dominant CaDHA morphology at all concentrations.

**Figure 9:**
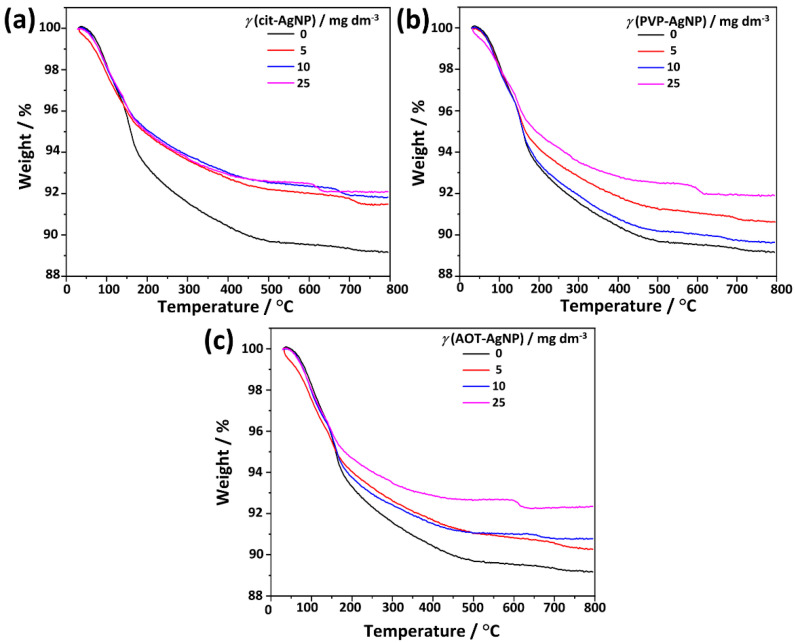
TGA curves of the precipitates obtained after 60 min reaction time in the presence of different concentrations of silver nanoparticles (AgNPs) stabilized with (**a**) citrate (cit-AgNPs), (**b**) poly(vinylpyrrolidone) (PVP-AgNPs), and (**c**) sodium bis(2-ethylhexyl) sulfosuccinate (AOT-AgNPs). *c*(CaCl_2_) = *c*(Na_2_HPO_4_) = 4 · 10^−3^ mol dm^−3^, pH 7.4, 25 °C.

To determine if the incorporation of differently stabilized AgNPs influences the thermal behavior of formed precipitates TGA analysis was performed. From the TGA curve of the control system (Figure 9) can be concluded that the thermal decomposition of the precipitate, formed in the control system after 60 min, proceeds in four steps. In the first step, at temperatures between 50 °C and 140 °C, a weight loss of about 4.5% was observed. Two weight losses at higher temperatures of about 3% and 4.6% were observed in the temperature range of 140–170 °C and 170–500 °C, respectively. Weight loss of 0.3% was observed at temperatures above 700 °C. Previous studies have shown that the thermal decomposition of CaDHA proceeds in three steps. In the first step, at temperatures up to 130 °C, the weight loss is due to the loss of adsorbed water. At higher temperatures, two steps are observed that can be attributed to the decomposition of CaDHA [62,78,79,80]. On the other hand, a weight loss of adsorbed water can be observed in the TGA curve of OCP at temperatures around 125 °C [81]. The loss of crystallization water can be observed up to 300–350 °C, while the loss of chemically bound water occurs at temperatures up to 400 °C. At temperatures above 400 °C, a small continuous weight loss, corresponding to further water loss and decomposition, can be observed [82,83].

Previous studies have shown that the thermal decomposition of AgNPs usually proceeds in a dominant weight loss, attributed by the authors to the loss of water and organic components, at temperatures between 200–500 °C. The exact temperature range in which the dominant weight loss occurs depends on the type of AgNPs. Little or no loss was observed at temperatures above or below this range [84,85]. The TGA curves of the stabilizing agents used in the preparation of AgNPs are shown in Figure S3. The shape of TGA curves of the precipitates formed in the presence of differently stabilized AgNPs are similar to the one of the control system, i.e., weight losses characteristic for AgNPs and stabilizing agents cannot be discerned. However, several interesting features can be observed in the TGA curves of precipitates formed in the presence of differently stabilized AgNPs. Increasing the concentration of cit-AgNPS and AOT-AgNPs in the precipitation system resulted in a decreasing total weight loss. No such trend was observed in the presence of PVP-AgNPs (Figure 9). However, even in that case, the weight loss was smaller than in the control system. This confirmed the presence of AgNPs in the obtained precipitates. Moreover, weight loss was observed at temperatures above 600 °C, which was not present in the precipitate formed in the control system (Figure 9). The temperature at which the loss started decreased with increasing AgNPs concentration. Such weight loss was previously observed for crystalline apatitic CaP at 630 °C [86]. The observed differences in the thermal properties of precipitate formed in the control system and the presence of AgNPs are consistent with the observation that AgNPs present on the surface of β-TCP particles affect thermal stability [87].

**Figure 10:**
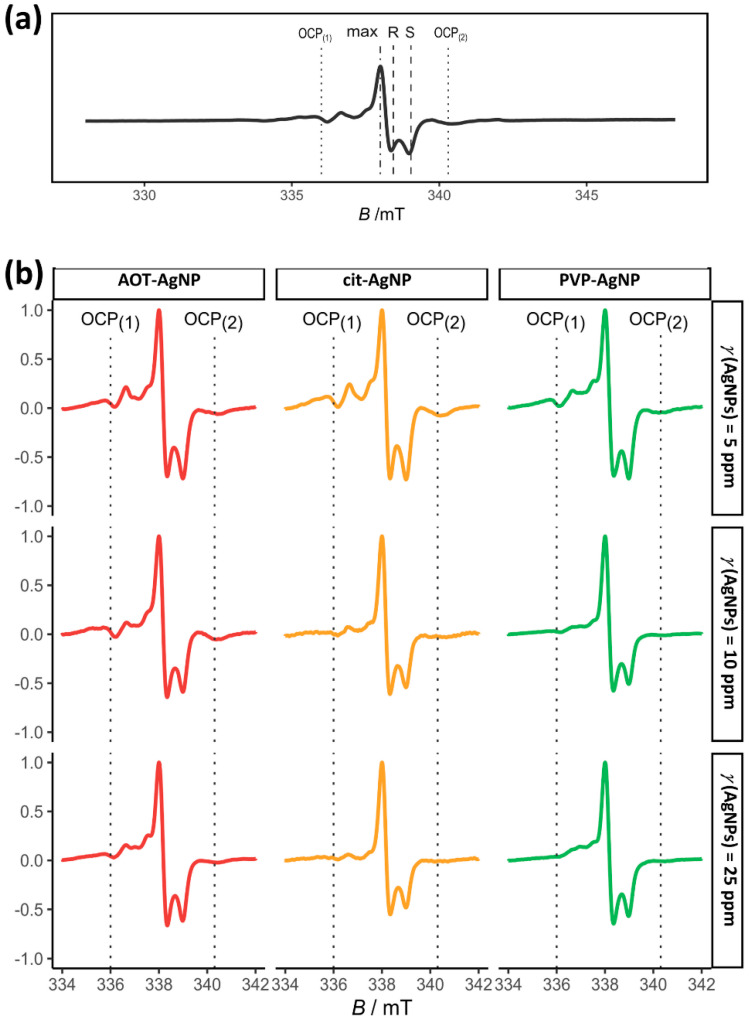
EPR spectra of (**a**) the precipitate obtained after 60 min reaction time in the control system and (**b**) the precipitates obtained after 60 min reaction time in the presence of different concentrations of silver nanoparticles (AgNPs) stabilized with citrate (cit-AgNPs), poly(vinylpyrrolidone) (PVP-AgNPs), and sodium bis(2-ethylhexyl) sulfosuccinate (AOT-AgNPs). *c*(CaCl_2_) = *c*(Na_2_HPO_4_) = 4 · 10^−3^ mol dm^−3^, pH 7.4, 25 °C.

In an attempt to determine more precisely the influence of AgNPs on precipitate composition EPR characterization was performed (Figure 10). The EPR analysis of the studied samples is quite complex. First, only powder spectra were available, which obviously provide less information than EPR spectra of single crystals. Second, the obtained spectra are composite and consist of multiple overlapping orientations averaged EPR spectra, i.e., spectral components in different ratios. Finally, special attention was paid to the method of determining the g-values. The fitting was not used because it is certainly unreliable for composite signals. Therefore, the phenomenological parameters, such as the *R*- and *S*-values, [88,89,90] were used to monitor the deviations in the line shape, i.e., the changes in the local ordering due to precipitation in the presence of AgNPs. The obtained differences in *R*- and *S*-values show the combined effect of changes in the chemical composition of the studied samples, differences in the microcrystalline orientation in different domains, and the competition of the relative contributions of the EPR spectral components isotropic, axial, or orthorhombic radical centers such as (${\mathrm{CO}}_{2}^{-},\;{\mathrm{CO}}_{3}^{3-},{\mathrm{CO}}_{3}^{-},{\mathrm{NO}}_{3}^{2-},\ldots .$).

The EPR spectra of the control sample clearly indicate that many paramagnetic centers are present in the sample in different ratios (Figure 10a). According to the literature data, [91,92,93,94] part of the spectral components can be attributed to the irradiated OCP, in terms of the specific EPR spectral features due to the presence of nitrogen radicals (two types of ${\mathrm{NO}}_{3}^{2-}$), the carbonate-centered stable ${\mathrm{CO}}_{2}^{-}$ radical. These two spectral features (denoted as OCP_(1)_ and OCP_(2)_ in Figure 10a) were not observed in the EPR spectra of CaDHA [61,62]. In addition, the EPR spectra of the control sample in the lower magnetic field position show a peak corresponding to a *g*-value of about 2.0115, probably a ${\mathrm{CO}}_{3}^{-}$ radical associated with entrapped water [95,96] and another type of carbonate-centered stable ${\mathrm{CO}}_{2}^{-}\;\mathrm{radical}$. Since the powder spectra of the two ${\mathrm{CO}}_{2}^{-}$ radicals are nearly identical, such direct identifications cannot be performed on powder EPR spectra. Therefore, from the EPR spectra of the control sample, it appears that a mixture of CaDHA and octacalcium phosphate OCP was formed. This finding is consistent with the X-ray, FTIR, and SEM analyses (Figure 7 and Figure 8).

For all three types of stabilizing agents, subtle differences in spectral shape can be observed. Table 2 shows the *R*- and *S*- values extracted from the experimental spectra. The values change for all samples studied due to the changes in concentration ratio the of different radicals originating from OCP and CaDHA. For PVP-AgNPs and AOT-AgNPs, the relative changes, *ΔR* and *ΔS*, compared to the values of the control samples are the lowest at 10 mg dm^−3^, while for citAgNPs they are lowest at 25 mg dm^−3^, which can be attributed to the nature of stabilizing agent, i.e citrate. The largest changes in *S*- and *R*-values are observed at 5 mg dm^−3^ for all samples studied, about 29–34% and 62–65%, respectively. In addition, the decrease in intensity of the OCP_(1)_ and/or OCP_(2)_ signals with increasing AgNPs concentration was observed (Figure 10b). All these differences indicate that the addition of different types of AgNPs at different concentrations affects the component ratio in the resulting mixture of CaDHA and OCP. i.e., decreased amount of OCP with increased AgNPs concentration, confirming the results of the X-ray, FTIR, and SEM analysis (Figure 7 and Figure 8).

## 4. Conclusions

In this work, the influence of differently stabilized AgNPs, namely cit-AgNPs, PVP-AgNPs, and AOT-AgNPs, on the formation and transformation of CaPs was investigated. The stability of ACP was influenced only in the presence of the lowest and highest concentrations of cit-AgNPs and AOT-AgNPs, respectively. However, in the presence of AgNPs the morphology of ACP was strongly affected, and the nature and extent of the effect depended on the type of AgNPs. It is interesting to note that in all cases, mostly single AgNPs distributed throughout the ACP were observed, indicating their good dispersion in the ACP matrix.

After 60 min of reaction time, a mixture of CaDHA and a small amount of OCP formed in the control system. The presence of the AgNPs affected the composition of the precipitates formed. PXRD and EPR data indicated that the amount of OCP decreased with increasing AgNPs concentration. For PVPAgNPs and AOTAgNPs, the relative changes of phenomenological parameters *R*- and *S*- values, i.e., change in complex radical structures of EPR spectra, compared to the values obtained in the control sample, were the lowest at 10 mg dm^−3^, while for citAgNPs they were lowest at 25 mg dm^−3^.

The observed effects on the rate of amorphous to crystalline phase transformation, as well as on properties of both amorphous and crystalline phases, indicate that differently stabilized AgNPs can have different effects, even if they have a similar size (PVP-AgnPs and AOT-AgNPs). Although the effects of nanoparticles and stabilizing agents were not separated, as interactions at the interfaces play a major role in precipitation, this points to the important role of the stabilizing agent.

The possibility to fine-tune the solid phase properties, in this simple way, could be of interest for the rapid and cost-effective preparation of CaP/AgNPs composites for biomedical applications.

## Figures and Tables

**Table 1:** The hydrodynamic diameter (*d*_h_) and zeta potential (*ζ*) of silver nanoparticles (AgNPs), stabilized with citrate (cit-AgNPs), poly(vinylpyrrolidone) (PVP-AgNPs), sodium bis(2-ethylhexyl) sulfosuccinate (AOT-AgNPs) suspended in anionic reactant solution (*c*(Na_2_HPO_4_) = 8 · 10^−3^ mol dm^−3^, *γ* (AgNPs) = 50 mg dm^−3^, pH 7.4) at 25 °C.

Sample	Peak I		Peak II		*ζ*/mV
	*d*_h_/nm	vol. %	*d*_h_/nm	vol. %	
cit-AgNPs	15.9 ± 2.0	59.7 ± 3.7	64.4 ± 2.6	40.3 ± 3.7	−40.2 ± 1,4
PVP-AgNPs	89.9 ± 1.0	100			−24.5 ± 1.6
AOT-AgNPs	84.8 ± 0.8	100			−59.9 ± 2.5

**Table 2:** *R*- and *S*-values of the powdered irradiated precipitates obtained after 60 min reaction time in the presence of different concentrations of silver nanoparticles (AgNPs) stabilized with citrate (cit-AgNPs), poly(vinylpyrrolidone) (PVP-AgNPs), and sodium bis(2-ethylhexyl) sulfosuccinate (AOT-AgNPs). *c*(CaCl_2_) = *c*(Na_2_HPO_4_) = 4 · 10^−3^ mol dm^−3^, pH 7.4, 25 °C.

Sample	*γ* (AgNPs)/mg dm^−3^	*R*	*S*
Control system	0	0.536	0.443
Cit-AgNPs	5	0.719	0.729
	10	0.611	0.542
	25	0.549	0.482
PVP-AgNPs	5	0.692	0.720
	10	0.577	0.508
	25	0.643	0.567
AOT-AgNPs	5	0.698	0.719
	10	0.643	0.589
	25	0.662	0.615

## Data Availability

Data is contained within the article or Supplementary Materials.

## Supplementary Materials

The following supporting information can be downloaded at: https://www.mdpi.com/article/10.3390/ma16051764/s1, Figure S1: FTIR spectra of phosphate band region and corresponding first- and second-order differentiated spectra of precipitates obtained after 10 min reaction time in the control system and in the presence of silver nanoparticles stabilized with citrate, poly(vinylpyrrolidone), and sodium bis(2-ethylhexyl) sulfosuccinate; Figure S2: FTIR spectra of phosphate band region and corresponding first- and second-order differentiated spectra of precipitates obtained after 60 min reaction time in the control system and in the presence of silver nanoparticles stabilized with citrate, poly(vinylpyrrolidone), and sodium bis(2-ethylhexyl) sulfosuccinate; Figure S3: TGA curves of the stabilizing agents used for the synthesis of silver nanoparticles.
