# Supplementary material for: Can Differently Stabilized Silver Nanoparticles Modify Calcium Phosphate Precipitation?
Source: Materials (Basel). 2023 Feb 21;16(5):1764. doi: 10.3390/ma16051764 (PMC10003846; doi:10.3390/ma16051764)
Supplement: Supplementary file 1 [file materials-16-01764-s001.zip › materials-2208425-supplementary.pdf]

materials

Supplementary information for

Can Differently Stabilized Silver Nanoparticles Modify Calcium Phosphate Precipitation?

Suzana Inkret [1], Marija Ćurlin [2], Kristina Smokrović [3], Nikolina Kalčec [4], Nikolina Peranić [4], Nadica Maltar-Strmečki [3], Darija Domazet Jurašin [1] and Maja Dutour Sikirić [1,*]

[1] Laboratory for Biocolloids and Surface Chemistry, Division of Physical Chemistry, Ruđer Bošković Institute, Bijenička c. 54, 10000 Zagreb, Croatia
[2] School of Medicine, Catholic University of Croatia, 10000 Zagreb, Croatia
[3] Laboratory for Electron Spin Spectroscopy, Division of Physical Chemistry, Ruđer Bošković Institute, Bijenička c. 54, 10000 Zagreb, Croatia
[4] Institute for Medical Research and Occupational Health, Ksaverska cesta 2, 10000 Zagreb, Croatia
* Correspondence: sikiric@irb.hr; Tel.: + 385-1-456-0941

Citation: Inkret, S.; Ćurlin, M.; Smokrović, K.; Kalčec, N.; Peranić, N.; Maltar-Strmečki, N.; Domazet Jurašin, D.; Dutour Sikirić, M. Can Differently Stabilized Silver Nanoparticles Modify Calcium Phosphate Precipitation? *Materials* **2023**, *16*, 1764. https://doi.org/10.3390/ma16051764

Academic Editor: Nikolaos Bouropoulos

Received: 24 January 2023
Revised: 17 February 2023
Accepted: 19 February 2023
Published: 21 February 2023

Copyright: © 2023 by the author. Licensee MDPI, Basel, Switzerland. This article is an open access article distributed under the terms and conditions of the Creative Commons Attribution (CC BY) license (https://creativecommons.org/licenses/by/4.0/).

Content of listed material

Figure S1. FTIR spectra of phosphate band region and corresponding first- and second-order differentiated spectra of precipitates obtained after 10 minutes reaction time in the control system and in the presence of silver nanoparticles stabilized with citrate, poly(vinylpyrrolidone), and sodium bis(2-ethylhexyl) sulfosuccinate.

Figure S2. FTIR spectra of phosphate band region and corresponding first- and second-order differentiated spectra of precipitates obtained after 60 minutes reaction time in the control system and in the presence of silver nanoparticles stabilized with citrate, poly(vinylpyrrolidone), and sodium bis(2-ethylhexyl) sulfosuccinate.

Figure S3. TGA curves of the stabilizing agents used for synthesis of silver nanoparticles.

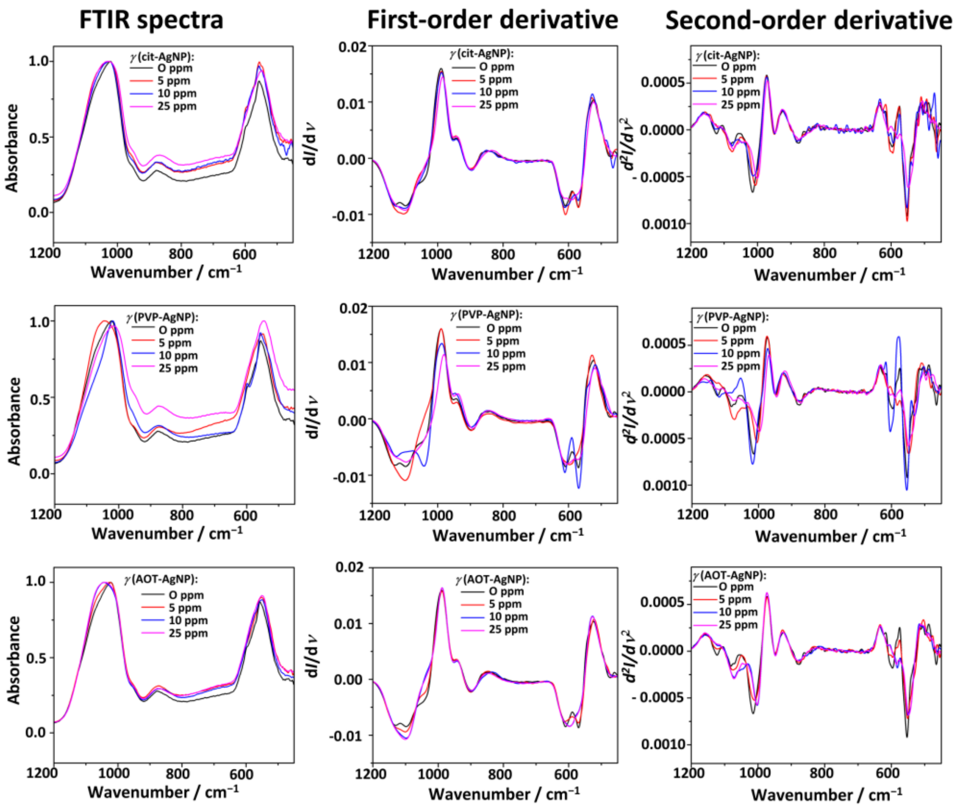

Figure S1. FTIR spectra of phosphate band region and corresponding first- and second-order differentiated spectra of precipitates obtained after 10 minutes reaction time in the control system and in the presence of different concentrations of silver nanoparticles (AgNPs) stabilized with citrate (cit-AgNPs), poly(vinylpyrrolidone) (PVP-AgNPs), and sodium bis(2-ethylhexyl) sulfosuccinate (AOT-AgNPs). $c(CaCl_2) = c(Na_2HPO_4) = 4 \cdot 10^{-3}$ mol dm^{-3}, pH 7.4, 25 °C.

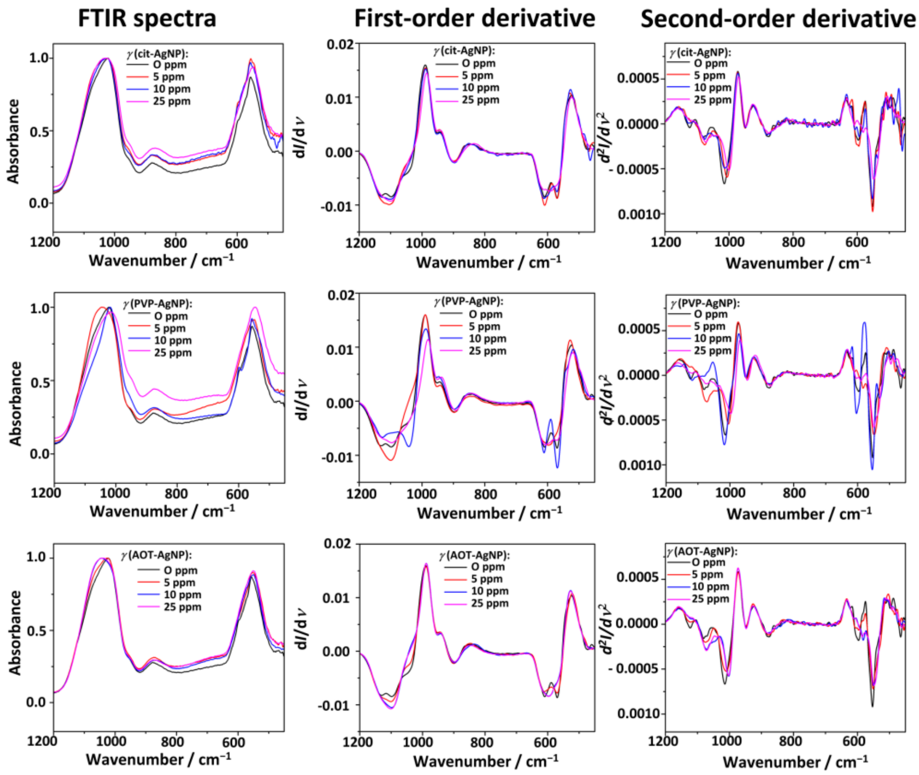

Figure S2. FTIR spectra of phosphate band region and corresponding first- and second-order differentiated spectra of precipitates obtained after 60 minutes reaction time in the control system and in the presence of different concentrations of silver nanoparticles (AgNPs) stabilized with citrate (cit-AgNPs), poly(vinylpyrrolidone) (PVP-AgNPs), and sodium bis(2-ethylhexyl) sulfosuccinate (AOT-AgNPs). $c(CaCl_2) = c(Na_2HPO_4) = 4 \cdot 10^{-3}$ mol dm^{-3}, pH 7.4, 25 °C.

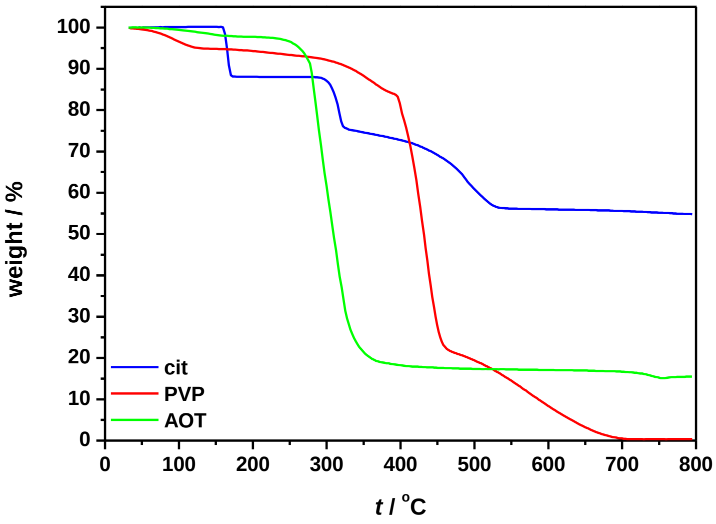

Figure S3. TGA curves of the stabilizing agents used for synthesis of silver nanoparticles: citrate (cit), poly(vinylpyrrolidone) (PVP), and sodium bis(2-ethylhexyl) sulfosuccinate (AOT).
